# Clinical laboratory shadowing- an elective program in undergraduate health professions training: perception, strengths and challenges

**DOI:** 10.1186/s12909-024-06355-5

**Published:** 2024-11-18

**Authors:** Vineetha K. Ramdas Nayak, Prajna P. Shetty, Vaideki Balamurugan, Vijetha Shenoy Belle, Ravindra Maradi, Kirtana R. Nayak

**Affiliations:** 1 Department of Physiology, Kanachur Institute of Medical Sciences, Mangalore, India; 2 Department of Biochemistry, Srinivas Institute of Medical Sciences and Research Center, Mukka, Mangalore, India; 3 Department of Biochemistry, Kasturba Medical College, Manipal, Manipal Academy of Higher Education, Manipal, Karnataka 576104 India; 4 Department of Physiology, Kasturba Medical College, Manipal, Manipal Academy of Higher Education, Manipal, Karnataka 576104 India

**Keywords:** Elective, Perception, Competency-based medical education

## Abstract

**Background:**

The authors aim to evaluate the perception, strengths, and challenges in the implementation of “Clinical Laboratory Shadowing” as an elective module for third-year professional medical students.

**Methods:**

Clinical Laboratory shadowing is an elective module designed by the faculty of Biochemistry for Phase III part-2 medical students. The elective comprises modules explaining the functioning of the Biochemistry Laboratory in a hospital. The students are provided insights into the different processes involved in laboratory testing. They are taught about total quality management of the Biochemistry Laboratory. Thirteen students from Batch 2019 and eight students from Batch 2020 volunteered to attend the elective. Students’ feedback regarding the learning was analyzed with the help of a questionnaire. Pre-test and post-test questions were evaluated to test the knowledge gained during the elective. Two focus group discussions were conducted regarding the benefits and areas for improvement for the elective.

**Results:**

Following the elective module there was a significant improvement in the test score compared to baseline. A majority (84–95%) of students perceived that the shadowing elective was well planned, and materials were well prepared, there was appropriate engagement with the facilitators, gained knowledge on the principles of total quality management, good laboratory practices, disease process, and diagnosis. Most (53–74%) of the students felt that this elective was not monotonous and not difficult. Around 84–95% of students felt that this elective module enhanced the importance of interprofessional communication and teamwork. Moreover, through focused group discussions, several recurring themes emerged. Among these, the scope for improvement within the module is profound. The faculties were of the opinion that the shift from conventional training to competency-based learning was effectively embraced by the students, underscoring the importance of consistent small group activities in the module to enhance the skills of Good laboratory practices. The students expressed a desire for additional clinical correlation and interpretation classes in the elective module, showing their preference for student-centred clinically oriented learning.

**Conclusion:**

The current study concludes that including an elective module on Clinical laboratory shadowing is an overall positive experience for medical students in enhancing their knowledge of basic sciences and application of the concepts in diagnosing cases. This elective may be desirable to adopt in other medical colleges in India.

**Supplementary Information:**

The online version contains supplementary material available at 10.1186/s12909-024-06355-5.

## Background

Medical education is advancing swiftly hence the National Medical Commission, India has improvised the undergraduate medical syllabus. The core curriculum ‘Competency-based undergraduate medical education’ curriculum denoted as CBME has been implemented by all Indian medical schools since the year 2019.The new curriculum puts a lot of emphasis on experiential learning. The CBME also envisions an undergraduate pupil as a potential scientist or researcher in the future. The fortune to have hands-on experience at the bedside, research laboratory, or community health care centers during the initial phases of student life serves as an instrumental experience for them as it will leave a positive impact on their future professional life. According to CBME, electives are compulsory and will be conducted for eight weeks, at the end of Final Professional Part I and the beginning of Part II with an aim to provide experiential learning to future doctors. A total of two hundred hours has been allocated for two sections of the elective course of four weeks each [1]. The students are given free will to choose from any of the modules offered by the medical school. An Elective is defined as “A learning experience created in the curriculum to provide an opportunity for the learner to explore, discover, and experience areas or streams of interest in the profession”. An elective facilitates students to explore the zone of growth and learning without any pressure of examination and emphasizes lateral thinking thus providing a future professional arena [1]. With reference to these guidelines, electives have been pioneered in the medical undergraduate curriculum in Indian medical schools.

Clinical Shadowing is a learning strategy in medical education where students are familiarised with the roles and duties of health care professionals [2]. It is a structured process in which a medical student witnesses a physician’s daily routine. The merits of shadowing for learners are dual: students witness the realities of their future profession as well as understand the level of expertise required [3]. Clinical practice is evolving and there are transitions in practice with few harsh realities of burnout, stress, and higher workload [4]. The students can figure out a way to be motivated and be aware of realistic situations. The advantage that clinical shadowing brings to the student is an observation of patient-doctor bonding, best practices, and techniques [5]. According to the literature students who have undergone clinical shadowing are more self-assured concerning their future goals and career selection [6–8]. One of the demerits reported in the literature is that the objectives of clinical shadowing are unspecific and students have to just observe and take notes [9].

The authors have designed an Elective module based on the same concept named “Clinical Laboratory shadowing” in which students understand “How does a Biochemistry Laboratory function”. The rationale behind this concept is to strengthen the co-relationship between basic science and clinical diagnostic skills as well as provide an insight into teamwork, leadership skills along interprofessional practices in healthcare.

To the best of our knowledge, clinical lab shadowing programs in India are rare and have not been implemented in various medical colleges. Clinical lab shadowing is a widely unexplored domain, especially in Indian settings.

## Method

### Research context and study design

A mixed method study was performed after obtaining ethical approval from the Institutional Ethics Committee (IEC1 35/2023) in two phases over a period of 1 year between December 2022–2023 inviting students from third professional year Part II of the Bachelor of Medicine and Bachelor of Surgery (MBBS) program of a medical college in South India. The yearly intake of students in the present medical college is 250, based on national eligibility entrance examinations. Competency-based curriculum is followed at the medical college where Biochemistry is taught in the preclinical phase. In the first academic year, the student understands the different diagnostic tests that are commonly done in clinical practice for assessing the normal functioning of the human body. In the next two years of academic training, the students train in paraclinical and clinical subjects. According to the Competency-based curriculum, an undergraduate medical student after the completion of the 3rd Professional year Phase I must compulsorily choose an “Elective” in the pre-clinical arena. An elective session lasts for two weeks. The study was conducted in the Clinical Biochemistry Laboratory. The clinical biochemistry laboratory is attached to a tertiary care hospital, and it is a NABL (National Accreditation Board for Testing and Calibration Laboratories) accredited laboratory.

### Study population

The study participants were the students belonging to Phase III Part 2 MBBS who had chosen “Clinical Lab Shadowing” as an elective for a period of 2 weeks. This study was conducted in 2 consequent batches of Undergraduate Medical Students.Totally 21 students participated in this study.

### Data collection methods

#### Features of designing of elective of ‘Clinical lab shadowing in biochemistry laboratory”

The elective session on “Clinical Lab shadowing” was conceptualized (VKRN) and delivered by faculty in Biochemistry (VSB, VBM, RM)during the academic year 2023–2024 comprehending the needs of undergraduate medical learners. The objectives of the module are mentioned in Table 1. The program was aimed to ensure that the sessions were conducted during elective sessions and did not interfere with the existing clinical classes. Relevant literature was referred to, and the clinical case scenarios to be addressed in relation to biochemistry in theory and practical modules were finalized.The specific learning objectives for each of the sessions were planned and the process or demonstrations to be done were documented.During each session of the elective, there was a briefing by faculty for one hour followed by interactive discussions with students.This was followed by practical demonstrations and observing various processes/procedures in the lab. A few sessions on clinical case scenarios and their laboratory diagnostic reports were also discussed. Faculty involved in teaching functioned as facilitators rather than experts. The sessions were conducted for two weeks in the afternoon for three hours.

**Table 1:** Representation of specific learning objectives of “Clinical lab shadowing” elective session

A. To know the types of vacutainers and mechanism of actions of anticoagulants
B. To understand and observe the different phases of the sample testing process with emphasis on laboratory errors.
C. To demonstrate the processing of the sample in the clinical laboratory
D. Hands-on experience in Biomedical waste disposal
E. To understand the basics of total quality management
F. To infer the results of common biochemical tests that aid in the identification of diseases in a supervised environment.
G. To understand and observe the reporting of critical values and amendments of reports.
H. To understand the importance of Lab accreditation
I. To function effectively as a team leader in a multi-disciplinary team managing a hospital-based laboratory
J. To learn communication with effective biochemical reasoning with peers and the health care team in the hospital
K. To know the ethical issues in laboratory management
L. To visit and observe the processing of the sample in the STAT lab.

External observers who were faculty from the Department of Biochemistry belonging to the same institution but were not involved in teaching in the given elective were invited for each session. The authors have collected their feedback on the content, delivery, and student participation during each session.

The strengths of the given elective session were analyzed using a semi-structured questionnaire (comprising open-ended questions and closed responses on a five-point Likert scale), and focused group discussions (FGDs) with students.

### Pre-test and post-test assessment

The authors devised a knowledge questionnaire consisting of 15 multiple-choice questions (MCQs) comprising one mark each. A pre-test was given on the first day of the elective and a post-test was given on the completion of 15 days of elective posting.

### Reflections from the students

A feedback questionnaire in English was provided to the students to assess their experiential learning. The semi-structured feedback questionnaire was filled out by all the students at the end of the given elective session. The questionnaire addressed the following: learning materials and outcomes, faculty engagement, implementation of elective module, and suggestions to improve the program. The feedback questionnaire was prepared (authors: VSB, VBM, RM, KN) and validated by a group of faculty members from departments of Physiology and Biochemistry from another college (VKRN, KJ, VJ). The feedback questionnaire was validated in a pilot study. The feedback questionnaire was ensured to be made available online for two working days. The student participants were informed in advance about the aims and implications of the medical education study. Participation was voluntary and consent was taken.

### Focus group discussion (FGD)

After all the students gave feedback by completing the questionnaire, the students were requested to participate in an FGD voluntarily. The questionnaire for the focus group discussion was prepared by the authors. The focus group discussion was moderated by faculty from Biochemistry (NM). Two Focus group discussions (FGD) were held with two successive elective batches to explore the student’s perception regarding the relevance and practicality of the elective module. Additionally, the authors wanted to provide a detailed analysis of their feedback and recommendations for enhancing the module.

The students who were willing to participate in an FGD voluntarily were invited. The FGD was digitally recorded in English by the note-taker (author VS). Verbatim transcripts were produced by author VS removing all the identifiers and later, verbatim transcripts were independently analysed by author PS.

### Statistical analysis

The pre and post-test scores were compared before and after attending the elective session using student t-tests. Frequency distribution was used to analyze student’s feedback questionnaires. The authors performed a thematic analysis of the focus group discussions conducted. The codes were analyzed to recognize patterns and themes.

## Results

Twenty-one medical students belonging to two different academic years of 2019 and 2020 participated in the study. There were 10 males and 11 females.

Amongst the students who attended the elective session knowledge scores which were (10.32 ± 2.11) at the pretest, showed a significant improvement (13.05 ± 1.51) at the post-test (Table 2). Most of the students perceived that the shadowing elective was well planned, and materials were well prepared, there was appropriate engagement with the facilitators, gained knowledge on the principles of total quality management, good laboratory practices, disease process, and diagnosis. The students felt that this elective was not monotonous not difficult and felt that this elective module enhanced the importance of interprofessional communication and teamwork (Tables 3 and 4).

**Table 2:** Comparison of pre and post-test scores before and after attending the elective session

	Scores (Mean ± SD)	*P* value*
**Pre-test**	10.32 ± 2.11	< 0.001
**Post-test**	13.05 ± 1.51	

*Student t test, p value less than 0.05 was considered statistically significant

**Table 3:** Feedback from students regarding their experiences during elective sessions of Clinical Laboratory shadowing

Sl.No.	Items	Strongly agree (*N*)	Agree (*N*)	Neutral (*N*)	Disagree (*N*)	Strongly disagree (*N*)
1	The elective module on early clinical laboratory shadowing was well planned and executed	11	7	1	0	0
2	The instructors involved in the module were well prepared (in terms of content, teaching methods, and activities)	14	4	1	0	0
3	The teaching materials provided were crisp and clear	10	6	2	1	0
4	The instructors involved were available to clarify any doubts and queries of the students.	10	8	1	0	0
5	Engagement with the faculties were appropriate for gaining knowledge about the subject	14	4	1	0	0
6	The elective modules helped me to understand a clinical laboratory’s role and importance in providing reliable results	11	6	2	0	0
7	The elective modules helped me to understand the principles of Total Quality Management (TQM) in laboratory	9	8	2	0	0
8	Active participation from the students is required to understand the principles of TQM	9	7	3	0	0
9	The elective modules helped me to understand good laboratory practices.	10	8	1	0	0
10	The laboratory visit helped me gain more knowledge on Total Quality Management	9	9	1	0	0
11	The interpretation of laboratory reports helped me improve my critical thinking and improved my understanding of a disease process	11	7	1	0	0
12	The elective module will aid me in the development of leadership skills and teamwork	9	9	1	0	0
13	The elective module will aid me in improving my interdepartmental and interprofessional communication skills	10	8	1	0	0
14	Did the elective module feel monotonous (for example, the repetitiveness of classes, the content of the module, teaching tools, laboratory activities, etc.)	0	2	2	11	14
15	Was the module content difficult to understand?	0	5	4	4	6
16	End-module examination was reflective of the course content	11	6	2	0	0
17	The elective module stimulated my interest in the subject and made me consider a diagnostic end specialty for my post-graduation	9	7	3	0	01
18	The learning environment was a safe space where I was encouraged to express myself without fear of judgment and ridicule	13	5	1	0	0
19	I would recommend this course to others.	11	6	1	0	1

**Table 4:** Student feedback to open-ended questions asked in the feedback questionnaire

“I would recommend my fellow colleagues to attend this elective. It was nice.”
“I personally hoped for a little more theoretical topics, I understand this is mostly lab related elective, but I liked the lab results interpretation class a lot because it helped in revisiting the basics which can also help in clinical subjects like medicine and strengthen our base subjects like biochemistry.”
“Everything was very well planned and up to the mark.”
“I missed few of the initial classes but the classes about quality assessment and analysis of lab reports were very helpful. Please increase the number of classes on inferring results of common scans as this class helped a lot in correlating diseases with the lab reports and giving us an idea about the actual clinical practice. Thank you. Had a very good experience here.”
“Interpretation of lab reports class should have been more.”

Recurring themes generated from both student faculty feedback (Tables 5 and 6) and were actually a blend of both deductive and inductive codes from grounded theory, that user engagement, knowledge retention, ease of use, applicability to quality clinical services are the milestones for the success of any training module.

**Table 5:** Students perception regarding the module

Themes	Categories	Inductive codes
General learning experience	The transition from phase 1 to 2 and 3	Inclination towards clinics Teacher centred training to very well student centred clinically oriented learning
Clinical Diagnostic skill	Clinical Diagnostic Skill (CDS) Development	Module Development
	Knowledge gain	Knowledge regarding calibrators, Quality control (QC), sample quality
	Lab report interpretation Different and suitable approach	Interpret the report precisely despite the reportable incidents (insufficient sample or possible pre-analytical, analytical & post-analytical error), false negatives and positives Efficiency in interpreting. Idea about the applicability of external and internal QC
Communication skills	Confidentiality	Conveying the clinical report to the right stakeholder
		Improved Interdepartmental communication
Good laboratory practice	Knowledge gain- Improved post elective session	Confidence in diagnosis
	Knowledge gain- Individual responsibility	Learnt the role of each stakeholder and the importance of every one’s role from diagnostic lab side to clinical side
Merits and demerits of elective	Strengthening the knowledge gained in preclinics	Real time experience than relating everything to the knowledge gain long back from didactic lectures and books
	Improved Confidence	Correlating the clinical reports in diagnosing
	Different and suitable approach	Interpret reports with different perspective
		Right test for the suspected condition
Scope for the improvement	Inclusion of more interpretation classes	

**Table 6:** Faculty Feedback regarding clinical lab shadowing elective session

Themes	Categories	Inductive Codes
General teaching experience	Transition	Teacher-oriented learning to student-oriented learning Teach specified SLOs Early clinical exposure (ECE), Attitude, Ethics and Communication (AETCOM), certifiable skills, Self directed learning (SDL) Horizontal & vertical integration Negligence in traditional learning regarding the syllabus Positive use of technology for both teaching and assessment
	Challenges	Lack of awareness regarding the Importance of the preclinical subjects in the final patient management
		Less interactive Lack of background knowledge
		Unaware of basic terminologies
Communication skills	Activity-based learning	Include more Role plays, short video clips of case scenarios
		More weightage for assessment of communication skills
	Small group learning	Group of 5
Good laboratory practice	Strength	All aspects including ethics covered
	Challenges	Lack of motivation
Scope for the improvement	Inclusion of hands-on training. More activities for Communication skills- Activity-based learning and Workplace based assessment.	Some hands-on training rather than lecture series, some activities on troubleshooting, clinical correlation, and advisory services
	Quality Good Laboratory Practice	Importance of the preclinical subjects in the final patient management Allowable range of variation in reports
	Admission criteria	Enrolment for the elective should be based on self-interest
	Formative Assessment	Regular assessment from the teacher instead of comprehensive teaching
Merits	Inclusion of this elective	Importance of including basic lab-related training address the challenges of traditional learning

## Discussion

Medical students identify shadowing as an opportunity to discover their areas of interest [10]. Through Clinical Laboratory Shadowing, students were sensitized to the functioning of the laboratory and made aware of the roles and duties of professionals deputed in the laboratory. The students could identify the importance of laboratory tests in the screening & diagnosis of diseases, monitor the effectiveness of treatment & predict the future risk of complications, and understand the procedure of processing the samples. They could identify the contribution of the entire healthcare team including technicians, housekeeping staff, laboratory assistants, and information technology supporting staff. The students observed the way collaboration happens – mainly through active communication and teamwork. The students also understood the mechanism by which accurate laboratory testing aids in early diagnosis and benefits patient outcomes. Shadowing gives opportunities for students to get insights into teamwork especially since every health professional requires a support system. This elective also highlights protocols being followed in the laboratory which gives them a better understanding of patient safety. This elective also made the students understand the importance of discipline and the workload in a laboratory. They got an insight into daily work, experience, and responsibilities in this elective.The studies have suggested that shadowing is an excellent tool that can be used in medical curricula to highlight the importance of the roles and responsibilities of healthcare professionals. Shadowing provides insight into teamwork and communication for better outcomes [11, 12]. Clinical lab shadowing may be the first stepping stone in orienting undergraduate medical students to learn the process, communication, and teamwork in the tertiary care hospital.

At the end of this elective module, a post-test was conducted that showed a significant increase in knowledge scores compared to the baseline pre-test scores (Table 2). This score indirectly indicates the effectiveness of the shadowing elective module in the gain of knowledge [13].

Using a validated questionnaire, students’ perception of the shadowing elective module was obtained. A majority (84–95%) of the students perceived that the shadowing elective was well planned, and materials were well prepared, there was appropriate engagement with the facilitators, gained knowledge on the principles of total quality management, good laboratory practices, disease process, and diagnosis. Most (53–74%) of the students felt that this elective was not monotonous and not difficult. Around 84–95% of students felt that this elective module enhanced the importance of interprofessional communication and teamwork (Tables 3 and 4). A study by Vidja K et al., also concluded that the objectives of the electives were met, students agreed that the elective was creative, teamwork was necessary and faculty contribution was necessary for the success of the implementation of elective [14].

The elective “Clinical Laboratory Shadowing” has various benefits such as being simple to implement. A recurring theme that has emerged with regard to the merits of the elective is increased confidence in diagnosis which was gained through basic science correlation along with improvised knowledge of the right test for the disease and interpretation of tests with different perspectives. The authors interpret this as the right conditioning for the students as they can link the principle and rationale behind ordering the tests [15]. Another theme that is emphasized is the improvised precise interpretation of reports in medical students which is very beneficial as previous literature has highlighted the plethora of investigations accessible to the medical fraternity, which has resulted in a strong reliance on laboratory results among clinicians [16]. Recent literature proposes that medical students feel that when they enter residency, they lack adequate knowledge and skills [17, 18]. Hence electives like Clinical Laboratory shadowing can provide insight into real-time experience, confidentiality, improved interdepartmental communication and the role of stakeholders which have emerged as strong themes in the FGD (Tables 5 and 6).

The theme that has emerged from the faculty discussion is that the elective focuses student centred clinically oriented learning. The major challenges noted are lack of knowledge in basic sciences co-relation in patient management. One of the suggestions was to include a role play and similar such activities in the elective and formative assessment regarding the enhancement of communication skills (Table 6). Many authors propose activity based learning and assessment as an effective approach in medical education [19].

### Strengths and limitations

Our shadowing course offers an elective module for other institutions to implement. With the electives being a relatively new concept in the Indian medical curriculum our study is limited by the evidence that the effectiveness of modules was adjudged only on the feedback of the students. A main limitation of our project is that this shadowing elective was performed in a single institution only. Another limitation of the study is that the feedback could be affected by the Hawthorne effect that is students wanted to please their teachers. The students who had a keen interest in biochemistry had joined this elective and there could be some amount of selection bias. In addition, there is no longitudinal follow-up done, to measure the change in behavior for the long term. The prospective study designs with efforts to combine follow-up to assess the acquisition of skills in the long term. Future steps for improvisation of the given elective will be to incorporate suggestions given by the first cohort of undergraduate pupils.

## Conclusion

In conclusion, Clinical lab shadowing sheds light on the basics of biochemistry and laboratory processes. The modules in Clinical laboratory shadowing were well received by students and they could appreciate the importance of good communication skills and lab practices. Overall, feedback received from both students and faculty is suggestive that the proposed elective module is interesting, feasible, and reproducible.

## Electronic supplementary material

Below is the link to the electronic supplementary material.

Supplementary Material 1

Supplementary Material 2

Supplementary Material 3

## Data Availability

Data will be provided when requested.
