# Supplementary material for: Clinical laboratory shadowing- an elective program in undergraduate health professions training: perception, strengths and challenges
Source: BMC Med Educ. 2024 Nov 18;24:1324. doi: 10.1186/s12909-024-06355-5 (PMC11575193; doi:10.1186/s12909-024-06355-5)
Supplement: Supplementary file 1 — Supplementary Material 1 [file 12909_2024_6355_MOESM1_ESM.pdf]

Sl.No.	Items	Strongly agree	Agree	Neutral	Disagree	Strongly disagree
1	The elective module on early clinical laboratory shadowing was well planned and executed					
2	The instructors involved in the module were well prepared (in terms of content, teaching methods, and activities)					
3	The teaching materials provided were crisp and clear					
4	The instructors involved were available to clarify any doubts and queries of the students.					
5	Engagement with the faculties were appropriate for gaining knowledge about the subject					
6	The elective modules helped me to understand a clinical laboratory's role and importance in providing reliable results					
7	The elective modules helped me to understand the principles of Total Quality Management (TQM) in laboratory					
8	Active participation from the students is required to understand the principles of TQM					
9	The elective modules helped me to understand good laboratory practices.					
10	The laboratory visit helped me gain more knowledge on Total Quality Management					
11	The interpretation of laboratory reports helped me improve my critical thinking and improved my understanding of a disease process					
12	The elective module will aid me in the development of					

	leadership skills and teamwork					
13	The elective module will aid me in improving my interdepartmental and interprofessional communication skills					
14	Did the elective module feel monotonous (for example, the repetitiveness of classes, the content of the module, teaching tools, laboratory activities, etc.)					
15	Was the module content difficult to understand?					
16	End-module examination was reflective of the course content					
17	The elective module stimulated my interest in the subject and made me consider a diagnostic end specialty for my post-graduation					
18	The learning environment was a safe space where I was encouraged to express myself without fear of judgment and ridicule					
19	I would recommend this course to others.					
20	Any comments for improvement					
