# Supplementary material for: Clinical laboratory shadowing- an elective program in undergraduate health professions training: perception, strengths and challenges
Source: BMC Med Educ. 2024 Nov 18;24:1324. doi: 10.1186/s12909-024-06355-5 (PMC11575193; doi:10.1186/s12909-024-06355-5)
Supplement: Supplementary file 2 — Supplementary Material 2 [file 12909_2024_6355_MOESM2_ESM.pdf]

Faculty feedback

Introduction and consent:

Good evening everyone, I am Dr. _____________, Biochemistry would like to discuss your perception of the Elective module on early clinical laboratory shadowing. I would like to thank you for your participation in this discussion. The information will be kept confidential and only be used for improvising elective modules. The estimated time for this focus group discussion will be 20 mins.

All the information will be provided by all of you will be kept confidential. Your identity will not be disclosed to anyone.

This meeting will be audio recorded. Do we have permission to audio record this meeting?

We will be transcribing the Focus Group Discussion for qualitative analysis. We will be removing all identifiers and the data will be completely anonymized and, transcripts sufficiently redacted. We would also like to seek your permission for the open sharing of the data with educational researchers or with the journal or data publishing houses. Do we have permission for the data sharing?

Do you have any questions or clarification regarding this FGD before we further proceed?

Can you all please introduce yourself?

Introductory questions

Can you please tell me more about yourself?

Transition question/s

How was your experience of teaching and assessment of MBBS graduates as per CBME?

Key questions

1. “Can you tell me how was your experience of taking 1st early clinical lab shadowing for MBBS graduates?
2. “Can you tell us how can you improvise teaching communication skills? Are any changes required?
3. Any changes are required in teaching Good Lab practices improved after this elective module?
4. Any modifications needed in the topics covered in this elective?
5. Any changes required for the assessment?
6. What were the challenges you faced to engage students?
7. How were students’ interactions during the elective modules?
8. Any suggestions to improve this elective module?

Closing question/s

Are you interested to continue as faculty for the elective module for the next batch?

Thank you for your time.

End
