# Supplementary material for: Clinical laboratory shadowing- an elective program in undergraduate health professions training: perception, strengths and challenges
Source: BMC Med Educ. 2024 Nov 18;24:1324. doi: 10.1186/s12909-024-06355-5 (PMC11575193; doi:10.1186/s12909-024-06355-5)
Supplement: Supplementary file 3 — Supplementary Material 3 [file 12909_2024_6355_MOESM3_ESM.pdf]

Focus Group Discussion Guide

Introduction and consent:

Good evening everyone, I am Dr. _____________, Biochemistry would like to discuss your perception of Elective module on early clinical laboratory shadowing. I would like to thank everyone for your participation in this discussion. The information will be kept confidential and only be used for improvising elective module. The estimated time for this focus group discussion will be 30-40 mins.

All the information will be provided by all of you will be kept confidential. Your identity will not be disclosed to anyone.

This meeting will be audio recorded. Do we have permission to audio record this meeting?

We will be transcribing the Focus Group Discussion for qualitative analysis. We will be removing all identifiers and the data will be completely anonymized and, transcripts sufficiently redacted. We would also like to seek your permission for the open sharing of the data with educational researchers or with the journal or data publishing houses. Do we have permission for the data sharing?

Do you have any questions or clarification regarding this FGD before we further proceed?

Can you all please introduce yourself?

Introductory questions

Which batch do you belong to? Can you please tell me more about yourself?

Transition question/s

How has been your experience of learning and assessment in Phase I and Phase II?

Key questions

1. "Can you tell us how Clinical Lab shadowing has helped you in developing clinical diagnostic skills?
   Probes: use of quality control, sample quality check, calibration
2. "Can you tell us how Clinical Lab Shadowing has helped you to improvise your communication skills"
   Probes: communicating with ward sisters, Postgraduates, physicians, dealing with technicians
3. Has your knowledge and understanding with respect to Good Lab practices improved after this elective module
   Probes: universal precautions, lab safety procedures
4. Has your confidence with respect to diagnosing a disease improved after this elective?
   Probes: history and lab findings
5. What are the merits and demerits of Clinical Lab shadowing?
6. Do you need any changes in the delivery of the elective module?
   Probes: activity based, hands on
7. Do you think this experience of Clinical Lab shadowing help you in the future?

Probes: diagnosis of cases, requesting specific tests

Closing question/s

Do you need anything else to know about the lab?

Thank you for your time.

End
